# A novel shaped-controlled fabrication of nanopore and its applications in quantum electronics

**DOI:** 10.1038/s41598-019-55190-y

**Published:** 2019-12-09

**Authors:** Chien-Han Chen, Xuyan Chang, Cen-Shawn Wu

**Affiliations:** 1 Graduate Institute of Photonics, National Changhua University of Education, Changhua, 500 Taiwan; 2 Department of Physics, National Changhua University of Education, Changhua, 500 Taiwan

**Keywords:** Nanopores, Electronics, photonics and device physics

## Abstract

High-intensity (10^7^–10^8^ A m^−2^) electron beams can be used to fabricate nanoscale pores. This approach enables real-time observation of nanopore drilling and precise control of the diameter of the nanopore. Nevertheless, it is not suitable for tuning the nanopore’s sidewall shape. In this study, we demonstrate the use of low-intensity electron beams to fabricate nanopores on a silicon nitride (SiN_x_) membrane. This technique allows the precise adjustment of the nanopore dimension and the shaping of its three-dimensional (3D) nanostructure. The 3D structures of the nanopore were evaluated by electron tomography, and series of oblique images were used in reconstructing the 3D images of nanopores using a weighted back-projection method. The sidewall shape of the nanopore was observed at different electron-beam conditions, and the formation mechanism was elucidated based on these results. The nanopore fabricated with this technique can be used as a template to develop electronics at the nanoscale based on which a quantum-dot device can be prepared with a simple evaporation process. The measured results show that the device can resolve well-defined electronic states that are characteristic for the behaviors of the quantum-dot device.

## Introduction

In the past decade, solid-state nanopores have drawn extensive attention because of their capacity for use for rapid electronic signal and biomolecular detections, and for the tremendous prospect for use in the next generation of diagnostic devices in nanomedicine^1–11^. The reactive ion etching technology^12^ or the electron beam from a transmission electron microscope (TEM) can be used to construct nanopores on silicon nitride (SiN_x_)^13,14^ and silicon oxide (SiO_2_) membranes^15^. These two approaches demonstrate great repeatability and have been widely used in fabricating nanopores, but the method of choice is to drill them using an electron beam in a TEM^16,17^. The advantage of the use of an electron beam to make nanopores is that a) it can precisely control the size of the nanopore and b) it does not cause ionic contamination. These advantages are increasingly important as nanopores are used in the system of biomolecular detections. In the study of electron-beam drilling by Dekker *et al*.^18^, a high-intensity focused electron beam was used to drill nanopores on a silicon oxide membrane. This showed that the electron beam could be accurately controlled for drilling pores at the nanometer scale while the drilling condition was monitored.

In deoxyribonucleic acid (DNA) sequencing, the size of the nanopore is important^19,20^, whereas the sidewall shape constitutes another critical factor^21^. In this study, we used a low-intensity electron beam to create nanopores on a SiN_x_ membrane. This technique allows the precise adjustment of the nanopore dimensions, and creates its three-dimensional (3D) shape. The electron tomography is a powerful technique which has been used to characterize 3D nanoscale structures^12,22^. Accordingly, in this study, we investigated that the mechanism of nanopore sculpting with low intensity e-beam by employing electron tomography, and characterizing the drilling behavior of nanopores. Finally, we introduced a new manufacturing technology to prepare a metallic quantum-dot device. This device used the reconstructed nanopore as a template to deposit metal on its anterior and posterior sides to form a sandwich structure of the metallic nanopore. The measured results show that the device can be defined as a quantum-dot device with a discrete energy level that can be clearly observed.

## Experimental Details

The growth of low-stress amorphous silicon nitride membranes (thicknesses of 30, 75, and 110 nm) was carried out using the low-pressure chemical-vapor-deposition (LPCVD) method on a substrate with a thickness of 500 μm at a temperature of 780 °C immersed in ammonia and exposed to dichlorosilane gases. The thickness tolerance of the prepared membrane was approximately 10% and was measured by spectroscopic ellipsometry. Thereafter, photolithography and reactive ion etching (RIE) were used to form a square window with a side length of 750 μm on the silicon nitride membrane. This window was used as a mask for KOH wet etching. Given that the KOH was anisotropically etched on the silicon substrate, a silicon substrate with a pyramidal shape was formed. A square suspension of the silicon nitride membrane with a side length of approximately 30 μm was obtained on the silicon substrate notch.

A low-intensity electron beam (~${10}^{6}\,{\rm{A}}\,{{\rm{m}}}^{-2}$) from a Jeol 2010 TEM with an acceleration voltage of 200 keV was used to drill the nanopore on the silicon nitride membrane while the entire drilling process was monitored. The focused electron beam diameter can be defined by the spot size on the membrane upper surface, which had a diameter of approximately 3–5 nm. The nanopore drilling time was approximately 1–5 minutes, depending on the thickness of the membrane. TEM images can provide real-time feedback and allow control during the nanopore drilling processes. With *in-situ* electron microscopy, 2D imaging provides instant feedback for pore drilling. By using real- time image feedback, we could precisely and repeatedly manufacture the sidewall shape of the nanopore. In addition, we demonstrated the use of the low-intensity electron beam to fabricate nanoscale devices of arbitrary geometries on insulating platforms without the need for a mask or resist (see the Figs. S1–S3 of Supplementary Material). The IMOD software modeling program was used to reconstruct images to facilitate the evaluation of the 3D reconstructed images^23,24^. The shape of nanopore can be depicted and analyzed by 3D TEM tomography (see the Fig. S4 of Supplementary Material). Before reconstructing the 3D structure of the nanopore, a TEM equipped with a Gatan Ultrascan 2.4k × 4k charge-coupled device (CCD) camera was used to acquire a series of oblique images of the nanopore to provide adequate information for the reconstruction. A total of 122 TEM images were obtained from −30° to +30° at 1° increments with a dual-axis model. The 3D structure of the nanopore was reconstructed by calculating the weighted back-projection of a series of oblique images with the IMOD software. Finally, the electronic tomographic image had an in-plane (X–Y) pixel size of approximately 2.6 nm, and a size of approximately 0.35 nm along the Z–axis. Based on experience in reconstructing 3D tomographic images, it is inferred that the 2D microscope image is related to the sidewall shape of the nanopore.

## Results and Discussion

**Figure 1:**
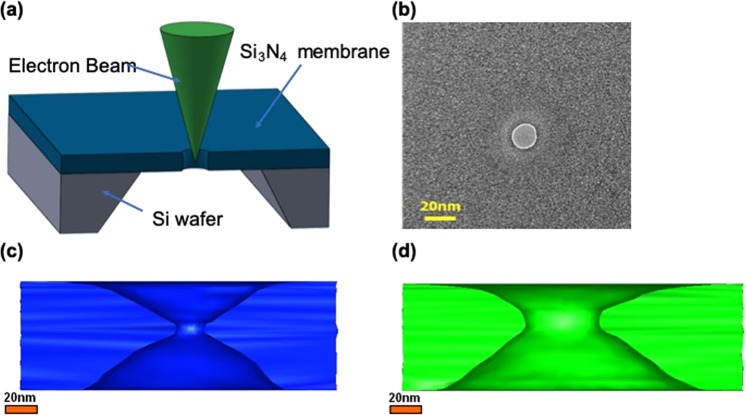
(**a**) Schematic of the drilling process of the nanopore with the use of a focused electron beam. (**b**) Transmission electron microscopy (TEM) image of a nanopore with a diameter of ~15 nm in SiN_x_ membranes with thicknesses of 75 nm. (**c**) Reconstructed three-dimensional (3D) image of nanopore with a diameter of ~15 nm in SiN_x_ membranes with thicknesses of 75 nm. Tomographic reconstruction is calculated by weighted back projection. The shape of the sidewall shape is hourglass. (**d**) 3D image of a nanopore with a diameter of ~50 nm in SiN_x_ membranes with a thickness of 75 nm.

Figure 1(a) shows a schematic of the electron beam used to drill nanopores on a silicon nitride membrane which was subsequently observed with TEM. When the high-intensity (${10}^{7}{\textstyle \text{-}}{10}^{8}\,{\rm{A}}\,{{\rm{m}}}^{-2}$) electron beam was used to drill nanopores on a silicon nitride membrane, a nanopore with an hourglass shape could be formed with sputtering^25–28^. The use of the high-intensity electron beam for the illumination of the nanopore narrowed the sharp edge of the hourglass shape to a cylindrical shape, and resulted in the destruction of the original shape^29^. By contrast, a low-intensity electron beam ($\sim \,{10}^{6}\,{\rm{A}}\,{{\rm{m}}}^{-2}$) could drill nanopores on a silicon nitride membrane without damaging the sidewall shape. Figure 1(b) shows an electron microscope image of a nanopore with a diameter of 15 nm. The 3D reconstructed image based on the use of a series of oblique photographs of the nanopore obtained by electron tomography shows that the nanopore has an hourglass shape. When the nanopore diameter changed from 15 to 50 nm at increased drilling time, the low-intensity electron beam which was used to drill the nanopore on the silicon nitride membrane did not destroy the original structure but maintained the hourglass shape. This indicates that the low-intensity electron beam was less destructive to the silicon nitride membrane during the drilling process, as shown in Fig. 1(c,d).

**Figure 2:**
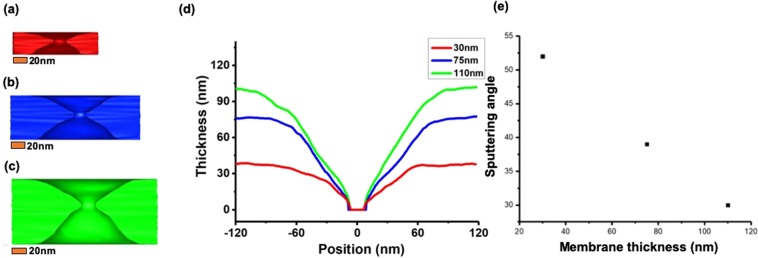
Reconstructed three-dimensional (3D) images of nanopores with diameters of ~15 nm in SiN_x_ membranes with thicknesses of (**a**) 110 nm, (**b**) 75 nm, and (**c**) 30 nm. (**d**) Plots of thickness vs. position. (**e**) Plots of thickness of membrane vs. sputtering angle *θ*.

To understand the relationship between the sputtering phenomenon and the thickness of the silicon nitride membrane, we used same electron beam conditions. Accordingly, the beam diameter was 5 nm and focused on the membrane during the nanopore drilling at different SiNx membrane thickness values. Figure 2(a–c) shows the focus beam drilled nanopore profile with 3D tomography. Figure 2(d) shows the correlation between the thickness and position using the reconstructed images. As the thickness and slope of the membrane increased, there was a correlation between the sputtering angle and membrane thickness. Figure 2(e) shows that the thicker membrane has a smaller sputtering angle *θ*. The hourglass shape of the nanopore sidewall shape could be explained by the sputtering model equation^30,31^,

1 $${E}_{{\rm{th}}}={m}_{0}{c}^{2}[{(\frac{M{E}_{d}}{2{{m}_{0}}^{2}{c}^{2}{\cos }^{2}{\rm{\theta }}}+1)}^{1/2}-1],$$

where *m*_0_ is the rest mass of the electron, *c* is the speed of light, *M* the atomic mass, *E*_*d*_ is the displacement or binding energy of the atom, and *θ* is the angle between the trajectory of the displaced atom and the direction of the incident electron. The value of the displacement energy (*E*_*d*_) of the SiN_x_ membrane is 16 eV, which corresponds to threshold electron energy (*E*_*th*_) of approximately 180 keV^25^. When the electron energy exceeded the threshold *E*_*th*_, it formed into an hourglass shape based on the sputtering on the anterior and posterior sides with the highest intensity of the electron beam applied at the center. The sputtering effect induced by the elastic scattering associated with the interactions among the electrons and atoms. The elastic scattering causes direct atomic displacement, such as the sputtering of the atoms^25^. Another mechanism of energy transfer from the incident electrons to the target material is radiolysis induced by the inelastic scattering^32,33^ associated with electron–electron interactions, resulting in the heating of the specimen^34,35^ and ionization. As the incident electron energy is increased, the effects of elastic scattering are increased, while those of inelastic scattering and heating are decreased. The main cause of the 3D sidewall shape of the nanopore was still dominated by the sputtering process.

**Figure 3:**
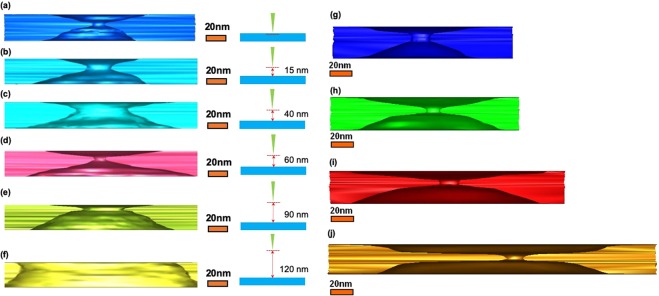
Reconstructed 3D images of nanopores. Use of different focal plane positions of nanopores drilled with electron-beams in a membrane with a thickness of 30 nm. Electron beam is focused with different heights, as follows: (**a**) 0, (**b**) 15, (**c**) 40, (**d**) 60, (**e**) 90 and (**f**) 120 nm. Reconstructed 3D image of nanopore sculpted by extended electron beam with diameters of (**g**) 20, (**h**) 40, (**i**) 80, and (**j**) 100 nm.

**Figure 4:**
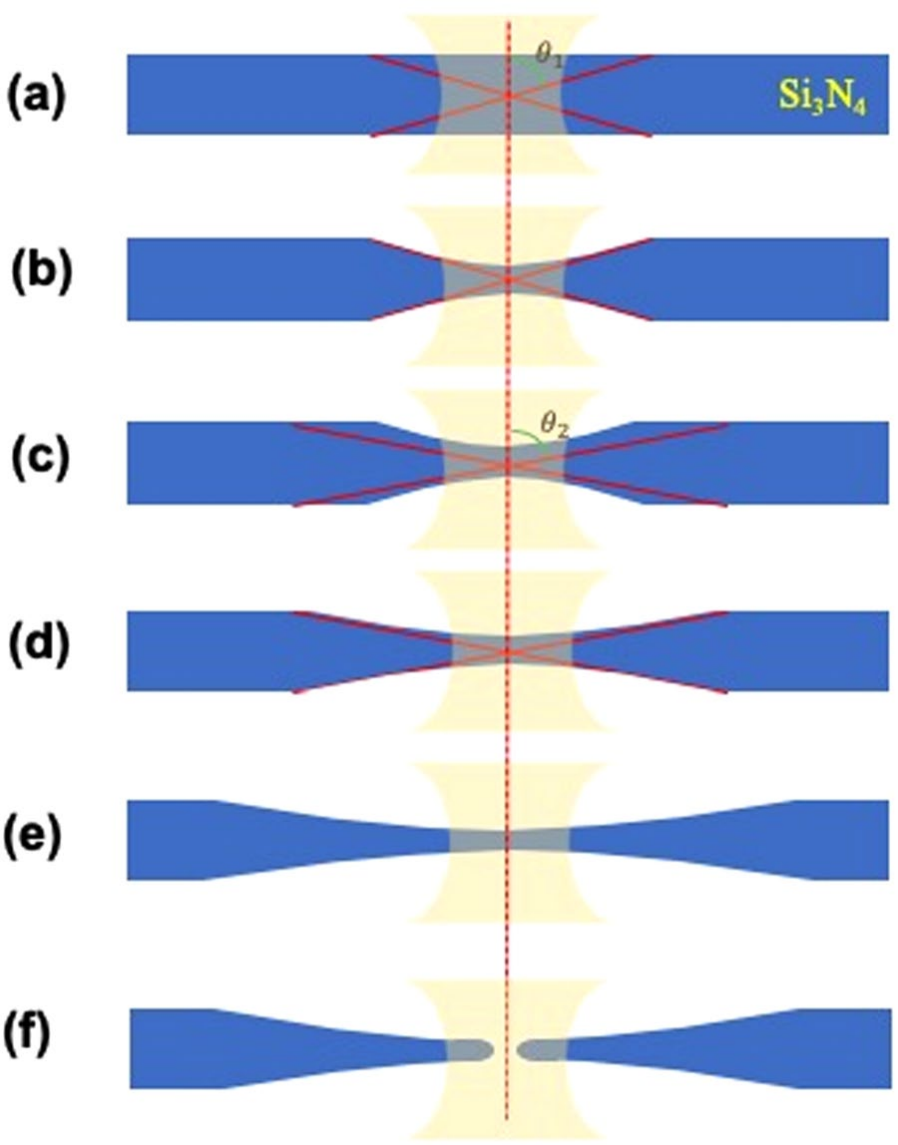
Schematic of nanopore drilling process using an electron beam with a diameter of 100 nm. Membrane thickness: 30 nm, beam intensity: 10^6^ A m^−2^, Focal plane: On the membrane surface. (**a**) Drilling of the SiN_x_ membrane with an electron beam with a diameter of 100 nm. The thickness of the membrane is large at the beginning, so the sputtering angle is relatively small. (**b**) Membrane thinning following the sputtering of the atoms. (**c**) Electron beam drilling is continually used, and the sputtering angle *θ*_2_ is larger than *θ*_1_ owing to the thinning of the membrane. (**d**) The membrane thickness decreases even more by the sputtering. (**e**) This cycle is repeated, eventually resulting in a flat and thin membrane. (**f**) Flat and thin membrane with a nanopore with an hourglass sidewall shape.

Because sputtering was the main cause responsible for the formation of the sidewall shape and started from the center of the electron beam, the adjustment to the focal plane was equivalent to the adjustment of the center position of the electron beam. This resulted in the adjustment of the nanopore shape of the focal plane. When the low-intensity electron beam was used in drilling the nanopore within the focal plane in different heights and to maintain the same beam condition which included the beam’s diameter and intensity, as shown in Fig. 3(a–f), it was found that the height of the narrow edge of the nanopore varied at different focal planes positions. This demonstrates that the hourglass shape associated with the beam profile depended upon whether the beam crossover or waist was inside or above the membrane. For drilling, we found that focus beams are more stable and made it easier to control the pore size compared with the defocus beam. Once a higher focal plane (defocus beam) is chosen for nanopore drilling, it is considerably much more difficult to control the drilled pore size. In conclusion, silicon nitride membrane damage from electron irradiation resulted in the direct atomic displacement that involved electron-atom scattering. When the impact of the electron-beam diameter on the drilling effect of silicon nitride membrane was investigated, electron beams with diameters of 20–100 nm were used. Changing the beam diameter from 20 nm to 100 nm would yield a beam intensity difference on the order of 10. The intensity of the electron beam was in the range of approximately 10^5^–10^6^ A/m^2^, which was focused on the membrane upper surface. It shows that if the diameter of the electron beam increased, the membrane of the nanopore becomes thinner and flatter, as shown in Fig. 3(g–j). Figure 3(j) shows that when an electron beam with a diameter of 100 nm was used to illuminate a silicon nitride membrane with a thickness of 30 nm for approximately 30 min, the membrane was relatively thin in comparison to the beginning. The underlying formation mechanism can be explained using the sputtering model, as shown in Fig. 4.

When an electron beam with a diameter of 100 nm was used to drill nanopores on a silicon nitride membrane with a thickness of 30 nm, a small sputtering angle *θ*_1_ could form owing to the large thickness of the membrane. The membrane became thinner after the atoms were sputtered. When the electron beam was continually used to drill nanopores, the thickness of the membrane was reduced and resulted in a larger sputtering angle *θ*_2_. Correspondingly, the thickness of the membrane was further decreased by sputtering. This cycle was repeated until a flattened and thin membrane eventually formed with an hourglass-shaped nanopore. A flat and thin membrane with an hourglass-shaped nanopore is a better choice for DNA sequencing. This is attributed to the fact that when the DNA sequence passes through the nanopore, a smaller thickness which provides a shorter transport length critically affects the translocation speed and measured signal resolution. One base pair of length along the strand is approximately 0.34 nm. A smaller membrane thickness results in a higher resolution for DNA sequencing^36–38^.

**Figure 5:**
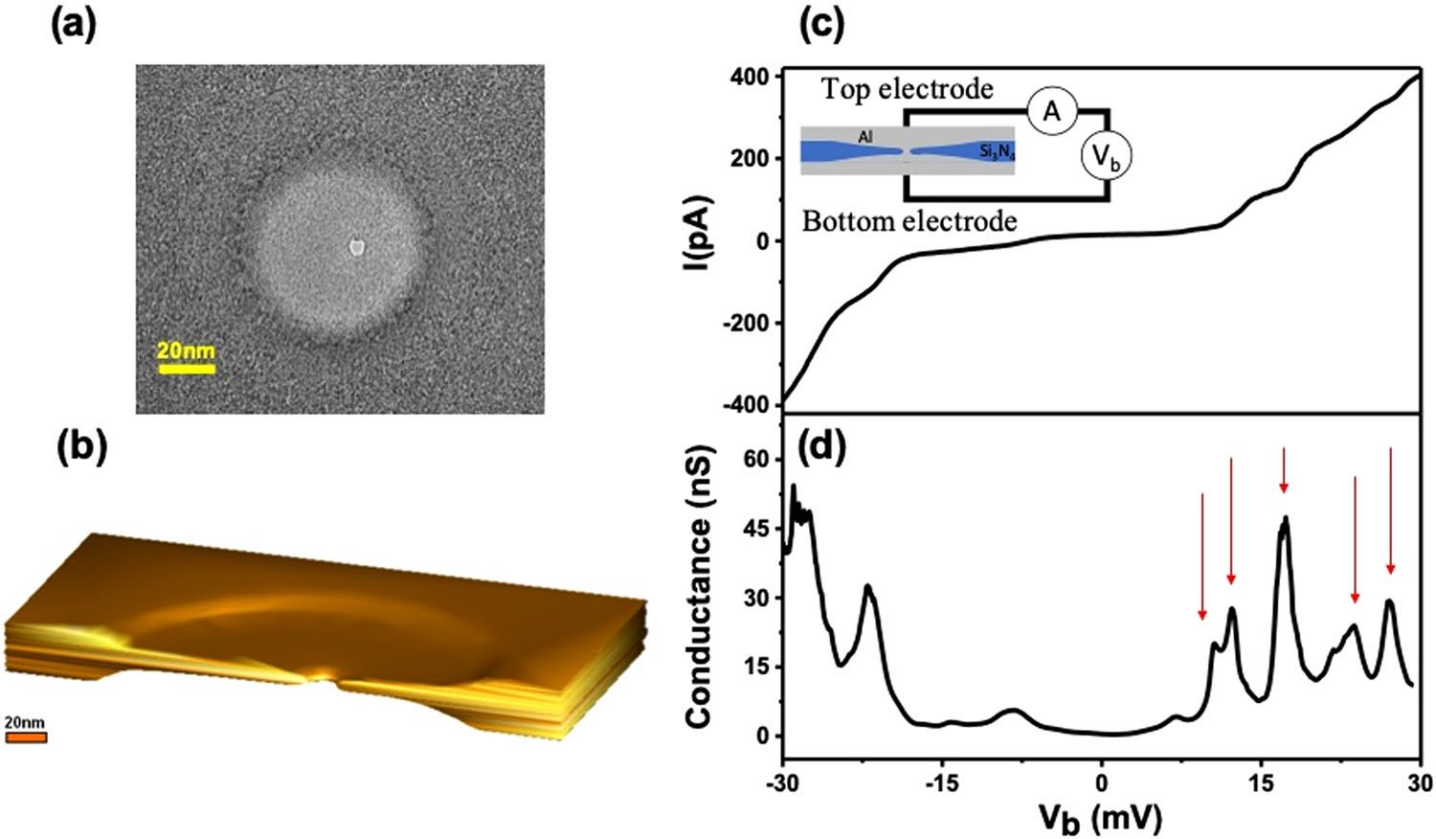
(**a**) Transmission electron microscopy (TEM) image of a flattened hourglass-shaped nanopore (**b**) Reconstructed 3D image of a flattened hourglass-shaped nanopore (**c**) *I–V* curve of device at *T* = 4.2 K. Inset: schematic of measurement setup of metallic quantum-dot device. (**d**) Differential conductance (*dI/dV*) as a function of bias voltage *V*_*b*_ at 4.2 K. The arrows denote the discrete electric states of quantum dot.

In addition to their applications in DNA sequencing experiments, a flattened hourglass-shaped nanopore can be used as a template for nanoscale electronic devices (see Fig. 5(a,b)), such as quantum-dot devices. To verify the conduction characteristics of aluminum used in the following experiments, preliminary experiments were carried out using a typical hourglass-shaped nanopore with a radius of 60 nm as a template (see Fig. S5 of Supplementary Material). The top part of the silicon nitride membrane of the flat nanopore in our experiment was vapor-deposited with 60 nm of aluminum to construct the upper electrode, and 250 nm of aluminum was vapor-deposited at the bottom to construct the lower electrode. A sandwich-structure device with a metallic disc was eventually achieved, as shown in the inset of Fig. 5(c). The experiments included the measurement of the tunneling current which flowed vertically from the upper electrode, via the aluminum’s nanoconstrictions, to the lower electrode. The samples were characterized by the measurement of the electrical transport properties at 4.2 K. Figure 5(c) shows the current–voltage (*I*–*V*) characteristics of the flat nanopore device. The *I*–*V* curve shows a suppression of the current at low voltages owing to the *Coulomb blockade* and stepwise increase of the current owing to the discrete energy spectrum. Additional step increases occur when the voltage is raised sufficiently to allow tunneling via other, high-energy discrete energy levels. Thus, a plot of differential conductance as a function of the bias voltage (*V*_*b*_) displays a series of peaks, as shown in Fig. 5(d). A large gap is observed, followed by a series of peaks associated with tunnelling into the excited states of the metallic quantum dots. This is an important experimental tool for investigating quantum dots where a gate electrode cannot be defined.

The spectrum of discrete electric states shown in Fig. 5(c,d) can be explained by the theory of metallic quantum dots^39,40^. When an electron enters the nanoconstriction region of the nanopore, an additional, nonzero energy can block the tunneling of electrons. If the applied bias voltage provides adequate energy to surpass the charge energy and overcome the discrete energy level difference, the electron can tunnel from the upper electrode on the dot. At this time, the electrochemical potential of the quantum dot will be enhanced, and the next electron will be blocked from the quantum dot. This phenomenon is called *Coulomb blockade*. The *Coulomb blockade* can be removed by changing the bias voltage. When the external bias voltage is further increased, and the energy corresponds to the discrete zero-dimensional (0D) states of the quantum dot, the Coulomb staircase effect can be observed. Accordingly, the position of the staircase will correspond to the positions of the 0D states. The structure of the device is similar to metallic point contact, but the experimental results have not observed the conductance quantization^41^. We provided an explanation based on which the deposition of the aluminum on this nanopore structure will form an electrically isolated particle rather than a metallic point contact. The conductance of the isolated particle can be measured by attaching it to electrodes via high-resistance tunnel junctions. From the positions of the voltage thresholds for steps in the *I*–*V* characteristics, the capacitances of the tunnel junction within the device can be determined directly^42^. The electrode-to-particle capacitances are *C*_1_ = 10.6 aF and *C*_2_ = 8.5 aF, and the charging energy is 4.2 meV. Based on the electrode-to-particle capacitances, the size of the particle can be roughly estimated; the radius of the particle is 3.0–3.4 nm by assuming it is spherical for the device^39^. In addition, the device has high-resistance tunneling junctions that provide weak coupling to the environment, the discrete electronic states can be observed. Finally, a simple sandwich structure constructed on this methodology enabled us to easily observe the behaviors of the metallic quantum-dot device. This device is expected to become a niche area in applications of quantum computing^43^.

## Conclusions

In summary, we demonstrated the low-intensity electron beam technique used to drill nanopores on a silicon nitride membrane, and exploited electron tomography to investigate the formation mechanism of the nanopore. We found that the low-intensity electron beam could maintain the nanopore’s shape without destroying its structure. When the focal plane of the electron beam was adjusted, the height of the narrow region of the nanopore changed. Accordingly, while the diameter of the electron beam was altered, the thinner membrane with the nanopore could be manipulated. The low-intensity electron beam had high elasticity, low destructiveness, and could also flexibly construct the shape of the desired 3D sidewall. Moreover, we presented a simple, versatile technique for the fabrication of quantum devices. This technology was used in conjunction with nanopore drilling and coating technologies to prepare metallic quantum-dot devices, and could be applied to the development of quantum electronics in the future.

## Supplementary information

Supplementary Material
