# Supplementary material for: A novel shaped-controlled fabrication of nanopore and its applications in quantum electronics
Source: Sci Rep. 2019 Dec 9;9:18663. doi: 10.1038/s41598-019-55190-y (PMC6901593; doi:10.1038/s41598-019-55190-y)
Supplement: Supplementary file 1 — Supplementary Material [file 41598_2019_55190_MOESM1_ESM.pdf]

Supplementary Material

A novel shaped-controlled fabrication of nanopore and its applications in quantum electronics

Chien-Han Chen[a], Xuyan Chang[b] and Cen-Shawn Wu[a, b*]

[a] Graduate Institute of Photonics, National Changhua University of Education, Changhua 500, Taiwan

[b] Department of Physics, National Changhua University of Education, Changhua, 500, Taiwan

Low-intensity electron beam drilling technique

Herein, we demonstrated the use of the low-intensity electron beams to fabricate nanoscale devices with arbitrary geometries on insulating platforms without the need of a mask or resist. *In situ* processes using TEM image monitoring are promising technologies for fabricating fine patterns and for reducing the processing steps. TEM images of example structures were used to demonstrate the flexibility of the low-intensity electron drilling technique.

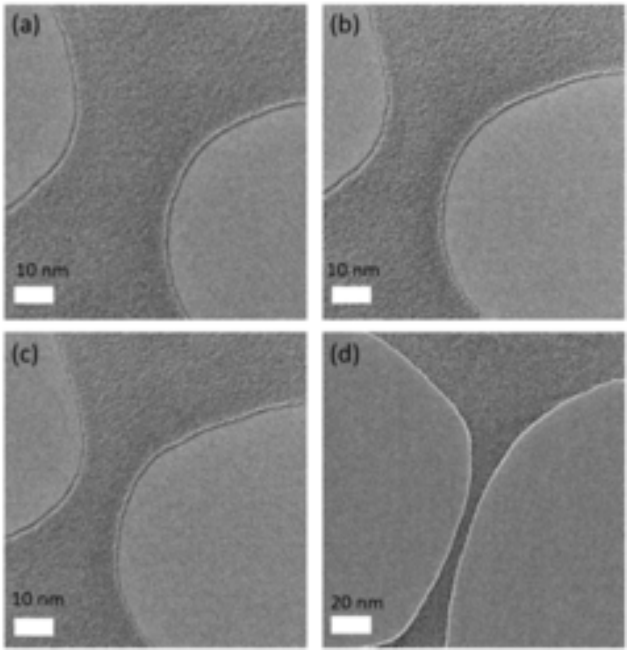

Figure S1. Sequence of transverse electron microscopy (TEM) images displaying the dynamics of the nanowire (NW) drilling process on the SiN_x membrane.

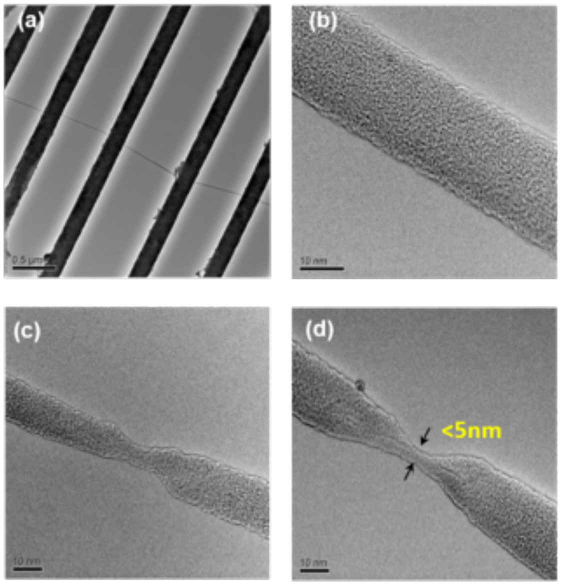

Figure S2. Sequence of TEM images displaying the dynamics of the drilling process of the suspended carbon nanowire. The nanowire [Fig. S2(d)] made of carbon NW with 5 nm width.

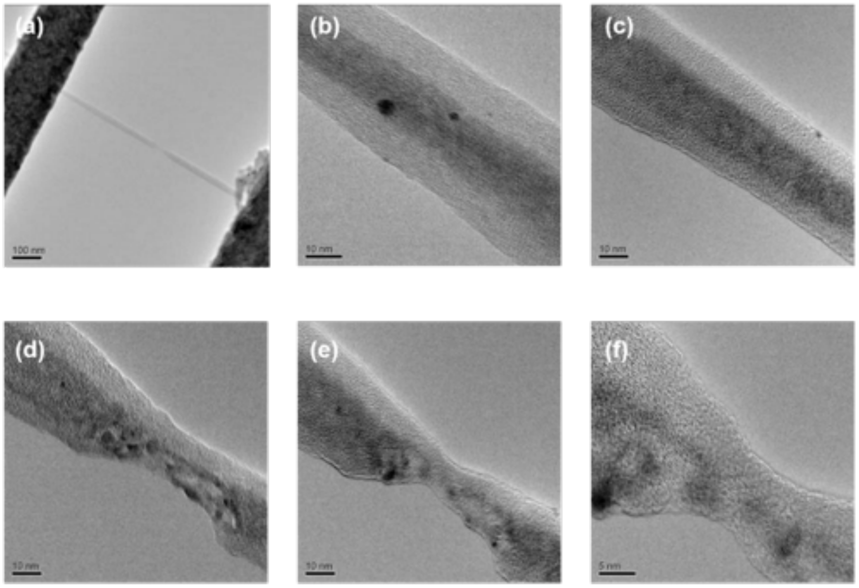

Figure S3. Sequence of TEM images displaying the dynamics of drilling the suspended multiwall carbon nanotube.

TEM tomography of the flattened hourglass-shaped nanopore

Electron tomography was introduced and has been used for decades for biological applications and in material science. A growing interest in electron tomography has been documented in recent years. The basic requirement for electron tomography is the assumption that the projected signal varies monotonically with the physical property of the sample. For amorphous materials, such as the SiN_x membrane, it can be assumed that this condition is fulfilled in the bright field TEM mode as the image contrast is dominated by elastic scattering. Electron tomography possesses a potential for 3D analyses of device structures at the nanoscale. However, there is an inherent limit to the range of tilt angles that leads to information loss in reconstructed volumes. Tilting around two axes is important for an accurate study of the 3D organization of amorphous nanostructures. The examples discussed herein show that by slicing through the reconstructed volume details more accurate metrology can be obtained compared to standard two-dimensional (2D) imaging.

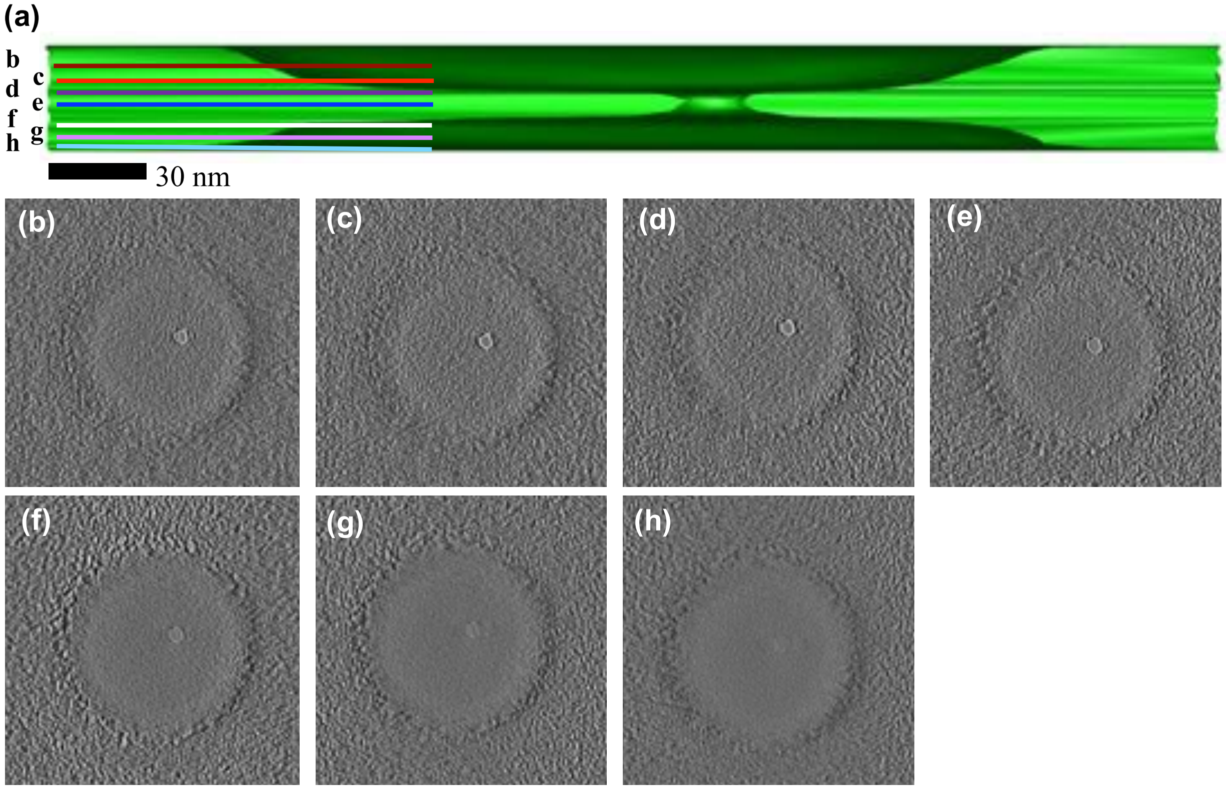

Figure S4. Three-dimensional structure of a 15 nm nanopore fabricated on a Si_3N_4 membrane. The resulting three-dimensional shape is shown in Fig. S4(a). Note that the sidewall of the pore has a flattened hourglass shape. The cutting positions of the seven slices through the membrane are indicated in panel (a).

Adjustable Superconducting S-c-S Junction

The typical hourglass-shaped nanopore with a radius of 60 nm can be used as the template for the superconducting S–c–S junction, where S and c denote the superconductor and the constriction, respectively. In the absence of an external magnetic field strength, the S–c–S junction becomes the superconducting nanocontact. The electrical transport properties of the superconducting nanocontact were measured. We found that a zero-voltage supercurrent should flow between the two superconducting electrodes separated by a nanoconstriction. For the sample with a maximal supercurrent of I_c = 2.3 μA, a superconducting energy gap Δ= 220 μeV at T = 150 mK was observed, as shown in Fig. S5(a). Type II superconductors allow different mixed states, where the magnetic field is allowed to penetrate along the nano-constriction through the material. This implies that the superconductivity state will be destroyed at the constriction by a critical magnetic field H_c. Fig. S5(b) shows the current–voltage characteristics of the S–c–S junction with the application of a perpendicular magnetic field. Our results indicate that the applied magnetic field generates vortices threading the nanoconstriction, and the dynamics of these vortices suppress the superconductivity. At this point, the S–c–S junction will become the S–N–S junction, where N denotes the normal metal. In addition, we found a strong magnetic field dependence of the zero-bias resistance (as shown in the inset of Fig. S5(b)), which is in agreement with the expectations of the Ginzburg–Landau theory. By controlling the magnetic field, we present a simple method to investigate an adjustable S–c–S junction system.

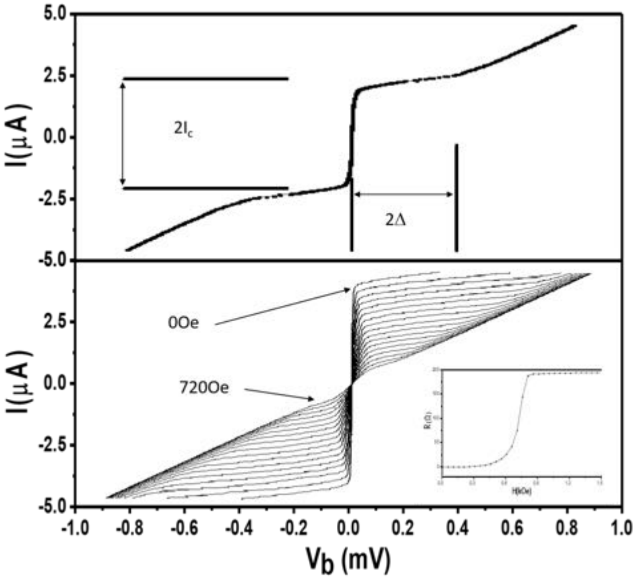

Figure S5. *I–V* characteristics of the Al nanoconstrictions at 150 mK exhibit superconducting behavior. The inset shows the magnetic field dependence of zero-bias resistance.
